# Volume Resuscitation in the Acutely Hemorrhaging Patient: Historic Use to Current Applications

**DOI:** 10.3389/fvets.2021.638104

**Published:** 2021-07-29

**Authors:** Kelly Hall, Kenneth Drobatz

**Affiliations:** ^1^Department of Clinical Sciences, Critical Care Services, Colorado State University, Fort Collins, CO, United States; ^2^Section of Critical Care, Department of Clinical Studies, University of Pennsylvania, Philadelphia, PA, United States

**Keywords:** hemorrhage, resuscitation, trauma, coagulopathy, shock

## Abstract

Acute hemorrhage in small animals results from traumatic and non-traumatic causes. This review seeks to describe current understanding of the resuscitation of the acutely hemorrhaging small animal (dog and cat) veterinary patient through evaluation of pre-clinical canine models of hemorrhage and resuscitation, clinical research in dogs and cats, and selected extrapolation from human medicine. The physiologic dose and response to whole blood loss in the canine patient is repeatable both in anesthetized and awake animals and is primarily characterized clinically by increased heart rate, decreased systolic blood pressure, and increased shock index and biochemically by increased lactate and lower base excess. Previously, initial resuscitation in these patients included immediate volume support with crystalloid and/or colloid, regardless of total volume, with a target to replace lost vascular volume and bring blood pressure back to normal. Newer research now supports prioritizing hemorrhage control in conjunction with judicious crystalloid administration followed by early consideration for administration of platelets, plasma and red blood during the resuscitation phase. This approach minimizes blood loss, ameliorates coagulopathy, restores oxygen delivery and correct changes in the glycocalyx. There are many hurdles in the application of this approach in clinical veterinary medicine including the speed with which the bleeding source is controlled and the rapid availability of blood component therapy. Recommendations regarding the clinical approach to volume resuscitation in the acutely hemorrhaging veterinary patient are made based on the canine pre-clinical, veterinary clinical and human literature reviewed.

## Introduction: Historic Approach to Hemorrhaging Patient

Acute hemorrhage in small animals results from traumatic and non-traumatic causes. While there are differences in overall patient management depending on the cause of the acute blood loss, systemic effects associated with volume of blood loss are repeatable. Co-morbidities associated with acute loss of intravascular volume and oxygen carry capacity are related to the proportion of blood volume lost and compensatory reserve of the individual patient (1, 2). While the physiologic response is predictable, resuscitation of the acutely hemorrhaging patient has dramatically shifted over the years, typically as a result of war-time medical efforts.

In human medicine, acute hemorrhage and its sequelae are the leading cause of preventable death in military and civilian settings (3). Over the last century, the approach to fluid resuscitation of the bleeding patient has gone through many iterations, frequently fueled by battlefield lessons, including whole blood transfusion (WWI), component therapy (WWII), synthetic and natural colloid fluids (Korean War), large volume crystalloid (Vietnam), back to whole blood (Iraq/Afghanistan conflicts) and coagulation testing guided component therapy (4, 5).

This review seeks to describe current understanding of the resuscitation of the acutely hemorrhaging small animal veterinary patient through evaluation of pre-clinical canine models of hemorrhage and resuscitation, clinical research in dogs and cats, and selected extrapolation from human medicine. It is recognized that mechanism-based reasoning for interventions in veterinary medicine predominate clinical decision making in the absence of large randomized clinical trials and systematic reviews of the clinical literature (6). Additionally, similar to austere environment challenges in human medicine, timing and availability of volume resuscitation resources in the acutely hemorrhaging small animal veterinary patient can vary significantly. Efforts to address these challenges and proposed solutions are addressed.

## Definition of Problem and Causes

For the purposes of this review article, patients experiencing acute blood loss are divided into 5 categories based on the cause of bleeding and the patient's hemostatic status upon arrival to a veterinary care facility (Table 1).

**Table 1:** Considerations for various manifestations of acute blood loss resulting in hemorrhagic shock.

	**Anatomic considerations for investigating blood loss**	**Clinicopathological parameters**	**Common manifestations**	**Approach**
Traumatic injury *Non-coagulopathic	External blood loss (e.g., oral, facial or skin wounds or lacerations) Internal blood loss - Gastrointestinal - Abdomen - Retroperitoneum - Thorax - Pericardium - Within solid organs (e.g., muscle, lung)	Packed cell volume increased (dogs), normal, decreased depending upon acuity a degree of hemorrhage Total protein/total solids: normal or decreased Lactate: increased (more likely in dogs than cats) BE: greater negative base excess paralleling lactate changes Decreased central venous oxygen saturation depending on severity PT/PTT: normal Platelets: normal to decreased	Blunt trauma (e.g., hit by vehicle, fall from height) Penetrating trauma (e.g., bite wounds, missiles)	Attenuate blood loss Crystalloids to ensure tissue perfusion with minimize dilutional and endothelial damage effects Ideal: replaced blood loss using PBC/Plasma/platelets in appropriate ratio or use fresh whole blood to replace? Colloids if low volume crystalloids ineffectual and blood product not available Active rewarming for patients without traumatic brain injury
Traumatic injury *Coagulopathic		Same as above except prolonged coagulation and/or abnormal viscoelastic monitoring parameters		Same as above. Correct acidosis with improving perfusion Active rewarming even more important Tranexamic acid?
Spontaneous bleeding (Non-traumatic) *Non-coagulopathic		Same as Traumatic injury non-coagulopathic	Splenic mass (e.g., hemangiosarcoma) Gastric ulceration Skin mast cell tumors	Same as trauma induced non-coagulopathic
Spontaneous bleeding (Non-traumatic) *Coagulopathic		Same as above Traumatic coagulopathic		Same as Trauma coagulopathic Vitamin K if rodenticide
Primary hemostatic disorder		Varies depending on underlying mechanism	Anticoagulant rodenticide (ACR) toxicity Immune-mediated thrombocytopenia Thrombocytopathias	Same as any blood loss hypovolemia Appropriate reversal or support of deficiencies, for example: Vitamin K (ACR) Platelets concentrates Treat Platelet dysfunction as indicated

### Traumatic Injury Causing Blood Loss, Non-coagulopathic

These are patients that have sustained injury from an external force (blunt and/or penetrating) leading to blood loss that can vary from minor to catastrophic. Source of blood loss can occur externally (e.g., lacerations, bite wounds), intracavitary (e.g., hemoabdomen, hemothorax), or into tissues (e.g., pulmonary contusions, crushing injuries). Primary intervention goals in these patients are to attenuate any ongoing bleeding not stopped by endogenous hemostasis (7, 8), appropriate and judicious volume expansion (based on patient's clinical response to volume of blood lost) and appropriately monitoring for development of consumptive coagulopathy and addressing co-morbidities associated with extravasation of blood (e.g., pulmonary contusions, cerebral hemorrhage).

### Traumatic Injury Causing Blood Loss, Coagulopathic

Trauma Induced Coagulopathy (TIC) is defined as the culmination of endogenous responses to hemorrhagic shock plus tissue injury (3). At presentation, TIC clinically manifests in a spectrum of phenotypes from hypocoagulable to hypercoagulable that impact decisions regarding appropriate initial intervention (3, 9–11). Individual patient's coagulopathic status can be dynamic throughout the resuscitation and resolution period as a result of ongoing blood loss, degree of endothelial injury, and interventions selected by the clinician. In these patients, primary intervention goals include those listed for non-coagulopathic traumatic bleeding, in addition to interventions based on diagnostic results (e.g., viscoelastic coagulation monitoring or specific hemostatic parameter measurement) and resources (e.g., whole blood, autotransfusion, component therapy) available to the clinician.

### Non-traumatic Spontaneous Bleeding, Non-coagulopathic

These are patients that are experiencing blood loss as a result of vascular defects caused by neoplasia (e.g., splenic hemangiosarcoma) or underlying disease (e.g., gastric ulcer, mast cell tumor). During primary survey, these patients can be a bit more challenging to identify as hemorrhagic shock until initial diagnostics are obtained. As such, in a patient showing signs of hypovolemic shock at presentation, initial intervention is typically volume expansion while preliminary diagnostics are performed to determine cause. If present, once shock is identified as hemorrhagic, intervention goals are similar to that of traumatic injury non-coagulopathic patients.

### Non-traumatic Spontaneous Bleeding, Coagulopathic

Similar to TIC, these are patients that have progressed to coagulopathy as a result of blood loss (without concurrent tissue injury). However, in contrast to TIC, these patients are identified as being in Disseminated Intravascular Coagulopathy (DIC) as a result of the consumptive processes associated with endogenous hemostatic efforts plus or minus contributions of endothelial damage due to underlying disease (12). Similar to non-coagulopathic, non-traumatic spontaneous hemorrhage, early identification of hemorrhage and source of bleeding are key. As with TIC, stabilization interventions are ideally based on diagnostic results (e.g., viscoelastic or specific hemostatic parameter monitoring) and resources (e.g., whole blood, autotransfusion, component therapy) available to the clinician.

### Primary Hemostatic Disorder

These are patients that are bleeding spontaneously from normal vascular “wear and tear” and have an inadequate ability for the endogenous hemostatic system to stop bleeding. Examples include thrombocytopenia (e.g., Immune-mediated thrombocytopenia, Ehrlichia), thrombocytopathia (e.g., Von Willebrand's Disease, medications), clotting factor deficiencies (e.g., hemophilias), or inactivity (e.g., anti-coagulant rodenticide). Primary intervention goals in these patients are to attenuate any ongoing bleeding which may require supplementation of endogenous hemostatic components that are deficient (e.g., platelets, plasma, cryoprecipitate), and based on patient's clinical status and degree of blood loss (Table 2), appropriate restoration of adequate oxygen delivery. An additional goal is to identify and administer therapies for those hemostatic defects that have a specific intervention that is able to prevent further bleeding (e.g., Vitamin K1 in anti-coagulant rodenticide toxicity).

**Table 2:** Signs and symptoms of hemorrhage by class as defined in the American college of surgeons advanced trauma life support 10th edition manual.

**Parameter**	**Class I**	**Class II (mild)**	**Class III (moderate)**	**Class IV (severe)**
Approximate blood loss	<15%	15–30%	31–40%	>40%
Heart rate	↔	↔/↑	↑	↑/↑↑
Blood pressure	↔	↔	↔/↓	↓
Pulse pressure	↔	↓	↓	↓
Respiratory rate	↔	↔	↔/↑	↑
Urine output	↔	↔	↓	↓↓
Glasgow Coma Scale score	↔	↔	↓	↓
Base deficit^*^	0 to −2 mEq/L	−2 to −6 mEq/L	−6 to −10 mEq/L	−10 mEq/L or less
Need for blood products	Monitor	Possible	Yes	Massive transfusion protocol

* *Base excess is the quantity of base (${\mathrm{HCO}}_{3}^{-}$, in mEq/L) that is above or below the normal range in the body. A negative number is called a base deficit and indicates metabolic acidosis*.

*↔Little to no change*.

*↓Decreased*.

*↑Increased*.

*Reprinted with permission from American College of Surgeons ATLS 10th Edition manual, Tables 3-1*.

## Physiologic and Pathophysiologic Considerations

In the continuum of advancing clinical care from bench to bedside, mechanistic models serve to describe the biological processes and physiology of a disease and/or the mechanism of action of an intervention. Utilization of the dog in preclinical and mechanistic models of hemorrhage is uncommon, but a number of published studies do exist (13–20). Model designs vary from fixed volume studies (removal of a set quantity of blood) to fixed pressure studies (removal of a quantity of blood to a set blood pressure). Other model variations include timing of hemorrhage, anesthetic protocol, addition of tissue injury, timing to intervention and type of intervention(s). These variations make broad generalizations across studies difficult. Despite differences in study design, in the hemorrhage phase of all studies, documented changes in instrumented, clinical and biochemical parameters have similar trends.

### Hemodynamic Changes Observed in Hemorrhagic Shock

**Figure 1:**
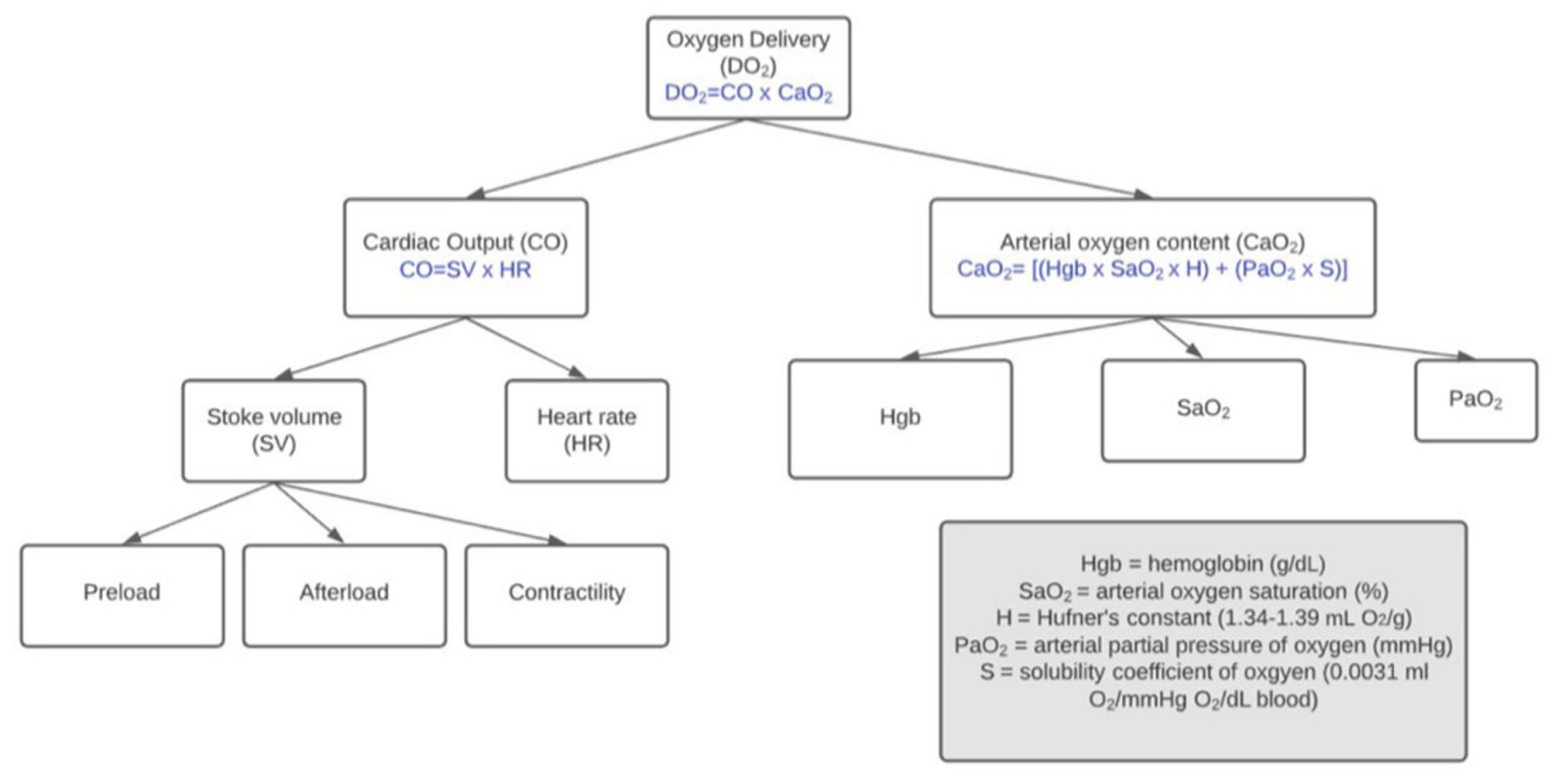
Factors affecting delivery of oxygen to tissues. During hemorrhagic shock (decreased DO_2_), the patient's physiologic compensatory mechanisms and clinician's intervention strategy relationships are interrelated.

The physiologic dose and response to whole blood loss in the canine patient is repeatable both in anesthetized (13–20) and awake animals (21). In addition to clinical and biochemical variables, these well-instrumented models measure cardiac index, central venous pressure, systemic vascular resistance, oxygen extraction ratios, oxygen delivery, and oxygen content. As progressive blood volume and oxygen carrying capacity from the intravascular space is lost, compensatory mechanisms come into play to attempt to maintain adequate delivery of oxygen to tissues (Figure 1). Decreased cardiac output due to decreased stroke volume (volume loss) is countered by activation of the renin-angiotensin-aldosterone-system (RAAS) and sympathetic nervous system leading to increased heart rate, contractility and systemic vascular resistance.

Decreased arterial oxygen content resulting from loss of hemoglobin is countered by mobilization of abdominal organ blood reserves into splanchnic circulation. In both dogs and cats, splanchnic circulation contributions to counteract blood volume loss is similar in total (~4.9 ml/kg cat; ~5.1 ml/kg dog). In dogs, splenic contraction is the biggest contributor whereas in cats, the contributions from liver, intestine and spleen are similar (14). In splenectomized dogs, a significant decrease in hemoglobin after acute hypotensive hemorrhage occurs; however, decreases in hemoglobin concentration do not occur in hemorrhaged dogs with an intact spleen (22, 23). In dogs, splenic contraction launches a significant number of red blood cells into the circulation. The net effect is a lesser impact of changes in hemoglobin concentration and oxygen carrying capacity compared to pre-hemorrhage levels in the pre-resuscitation phase of bleeding. Because arterial oxygen content may be less significantly impacted during acute hemorrhagic states, in dogs, the decrease in oxygen delivery is mainly attributable to a decrease in cardiac output (Figure 1).

Other systemic consequences and effects are dependent on the proportion of volume lost, duration of ongoing bleeding, and individual patient factors regarding compensatory reserve (Table 2). While impacted by the rate of loss, in dogs, cardiac arrest occurs when ~60–90% of blood is lost (24, 25) or mean arterial pressure (MAP) falls below 30–40 mmHg (13).

### Clinical Parameters (HR, SBP)

While an increase in heart rate (HR) and decrease in systolic blood pressure (SBP) are noted in canine models of hemorrhagic shock, compensatory mechanisms may result in these values remaining in the clinically normal reference interval until ~15–20% of total blood volume is lost (19). However, their relationship as represented by shock index (SI = heart rate/systolic blood pressure) may be an early indicator of compensatory shock, and requirement for volume resuscitation. Clinical literature and pre-clinical literature suggest that a SI > 1.0 at presentation warrants further investigation of volume loss and appropriate intervention (19, 26–28).

### Biochemical Parameters (pH, Lactate, Bicarbonate, Base Excess, Hemoglobin)

As blood volume is lost, compensatory mechanisms including buffering systems and ventilation adjust in an effort to maintain normal blood pH in the face of systemic metabolic acidosis characterized by significantly lower base excess and increases in lactate. The acidosis associated with acute hemorrhage can compound coagulopathy and result in continued bleeding (29). Volume resuscitation efforts to return appropriate delivery of oxygen and nutrients to tissues is key, and serial evaluation of base excess and lactate can help inform intervention decisions (16, 30, 31). While reduction in hemoglobin (Hgb) is noted with severe to catastrophic bleeding, with mild to moderate hemorrhage, prior to resuscitation, Hgb may be in the normal reference range. Clinically, normal Hgb at presentation does not rule out acute hemorrhage, and serial evaluation during volume resuscitation phase is useful.

### Coagulation

**Figure 2:**
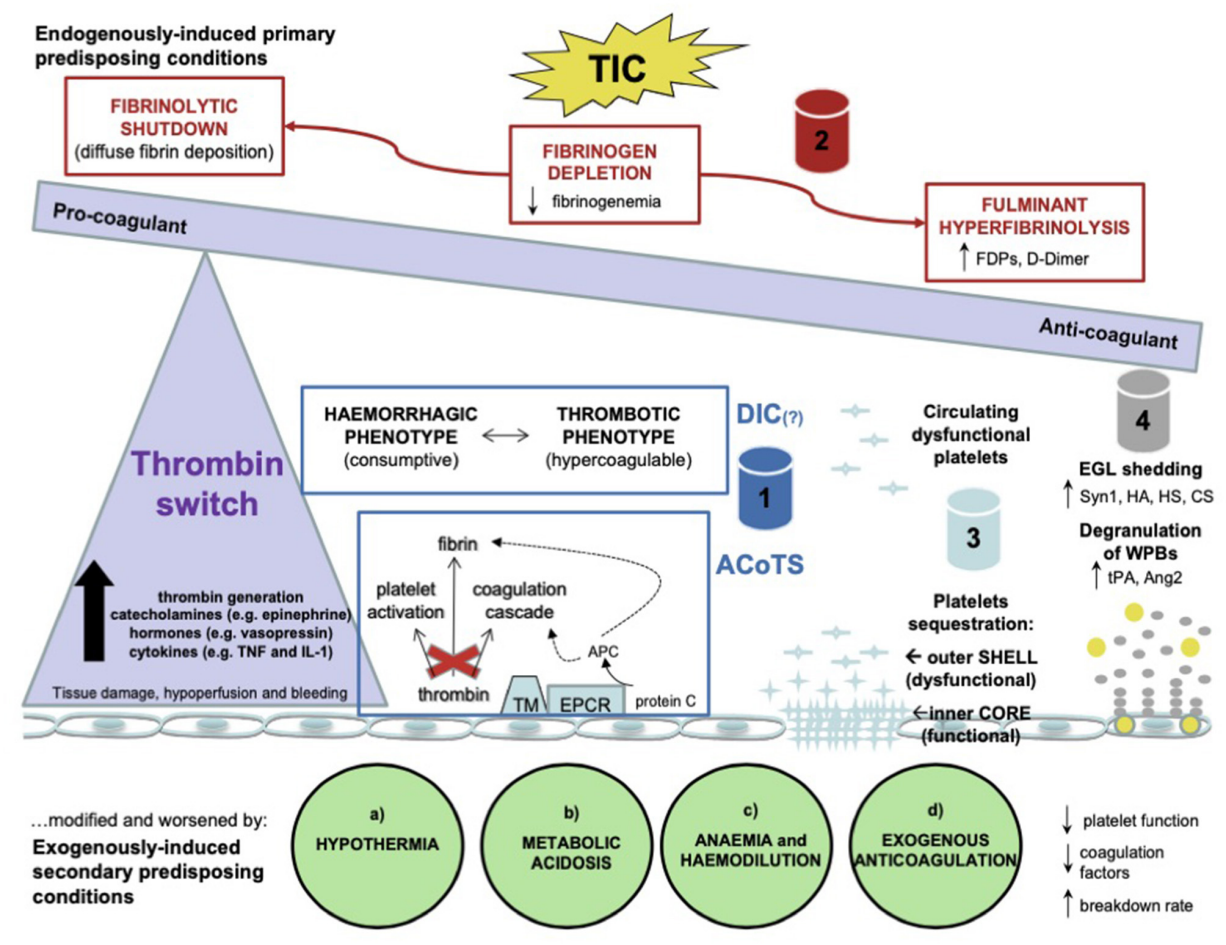
A schematic representation of the pathophysiological alterations in the hemostatic system occurring after trauma. Tissue damage, hypoperfusion and massive bleeding are the main drivers of the inflammatory and neurohormonal response with a significant increase in thrombin generation, catecholamines, hormones, and cytokines. Thrombin undergoes a systemic release which determines the so called “thrombin switch” toward anticoagulation. Trauma induced coagulopathy (TIC) is considered as a series of endogenously induced primary predisposing conditions based on 4 pillars: 1–endogenous anticoagulation in the form of disseminated intravascular coagulation (DIC) or acute coagulopathy induced by trauma and shock (ACoTS); 2–fibrinogen depletion, hyperfibrinolysis and fibrinolytic shutdown; 3–platelet dysfunction; 4–endotheliopathy. These conditions can be modified and worsened by exogenously induced secondary predisposing conditions in the presence of (a) hypothermia, (b) metabolic acidosis, (c) anemia and hemodilution, (d) exogenous anticoagulation. DIC, disseminated intravascular coagulation; ACoTS, acute coagulopathy induced by trauma and shock; FDPs, fibrinogen degradation products; TM, thrombomodulin; EPCR, endothelial protein C receptor; APC, activated protein C; EGL, endothelial glycocalyx layer; Syn1, syndecan-1; HA, hyaluronic acid; HS, heparan sulfate; CS, condroitin sulfate; WPBs, Weibel-Palade bodies; tPA, tissue plasminogen activator; Ang2, angiopoietin-2; PAR1, protease activated receptor 1; TM, thrombomodulin; APC, activated protein C; NO, nitric oxide; PGI_2_, prostaglandin I_2_; tPA, tissue plasminogen activator. Reprinted by permission from Springer Nature: Internal and Emergency Medicine, The current understanding of trauma-induced coagulopathy (TIC): a focused review on pathophysiology. Giordano et al. (32).

Hemostatic abnormalities are a major factor in the treatment of acutely bleeding patients, both in traumatic and non-traumatic hemorrhage. While there was a period of research that suggested that acute large volume hemorrhage in trauma was primarily associated with hypocoagulability and hyperfibrinolysis, growing evidence supports a continuum dependent on not only duration, amount of blood loss and clinician interventions, but also patient factors (Figure 2). This spectrum of hemostatic dysfunction is evident in pre-clinical canine hemorrhage research (33) as well as canine trauma clinical research (9–11, 34). In trauma patients, the added component of tissue injury, on top of blood loss, further clouds the picture, leading to various clinical manifestations of trauma induced coagulopathy (Figure 2). The body of evidence in human patient care regarding a spectrum of phenotypes associated with acute traumatic hemorrhage is growing (3). The advancement and development of bedside global hemostatic and fibrinolytic testing is enabling the ability to use blood product and resuscitation strategies based on individual patient parameters.

### Endothelium and Glycocalyx

Knowledge regarding the contributions of the endothelium and glycocalyx in injury and resuscitation is expanding rapidly (31, 32, 35, 36) (Figure 2). Mechanistic and clinical research to better describe the physiology and systemic response are expanding rapidly. As a result, there is clinical equipoise regarding “first choice” of volume resuscitation fluid for the acutely hemorrhaging patient in both human and veterinary medicine. Regarding the endothelium specifically, while no fluid is risk-free, there is evidence that crystalloid resuscitation may exacerbate endothelial and glycocalyx dysfunction and synthetic colloids are under continual review of their risks and benefits in hemorrhagic shock resuscitation (17, 33, 37) (*Cross reference Frontier articles: “The effects of resuscitative fluid therapy on the endothelial surface layer,” Smart/Hughes AND “Article 7 Colloids, Yes or No? Pros and Cons of Colloids?” (Adamik/Yosova)*. Large clinical trials evaluating alternative initial resuscitation fluid based on severity of disease and time to definitive care are underway in human medicine (38–41).

There is a broad range of clinical presentations of the acutely hemorrhaging veterinary patient as noted in section Definition of Problem and Causes. Understanding the physiology and mechanisms associated with acute blood loss help inform both clinical assessment and intervention decisions. In addition to patient variables (amount of blood lost, co-morbidities, hemostatic ability, compensatory reserve), resources available to the practitioner and pet owner influence the clinical approach to the acutely hemorrhaging veterinary patient. Despite a varied range of manifestations of the acutely hemorrhaging patient, a systemic approach as outlined below is recommended.

## Approaches to Fluid Resuscitation in the Medical Field

There has been a substantial change in human medicine regarding resuscitation for hemorrhagic shock from massive fluid administration to minimal fluid resuscitation while maintaining permissive hypotension and utilization of transfusion with a balanced ratio of blood products and goal directed correction of coagulopathy (3, 4). In some human hemorrhaging patient populations, plasma has replaced crystalloids for volume expansion (38–40).

For years, aggressive crystalloid therapy was utilized to treat hemorrhage shock. In fact, Shoemaker et al. recommended supra-normal resuscitation with crystalloids during shock. He found that this mode resulted in increased oxygen delivery to tissues and improved survival in critically ill patients (42). Unfortunately, this aggressive therapy resulted in generalized tissue edema, particularly bowel edema resulting inability to close the abdominal wall (43). Later, researchers demonstrated an association between supra-normal resuscitation and increased incidence of abdominal compartment syndrome, multiple organ failure, and decrease survival (44). Closer investigation into the consequences of supranormal resuscitation found compromised cellular function causing alterations in glucose metabolism and cardiac myocyte excitability (45–48). Subsequent studies have shown that aggressive crystalloid resuscitation can cause dilutional coagulopathy, acute respiratory distress syndrome (ARDS), multiple organ dysfunction syndrome (MODS), hypoxemia, compartment syndrome, endotheliopathy, and greater mortality (49, 50). Finally, subsequent randomized control studies utilizing supra-normal resuscitation did not demonstrate Shoemaker's initial successes (51).

Hypertonic saline is an attractive low volume resuscitation crystalloid. Its benefits include rapid increases in cardiac output and blood pressure (52), improved microcirculatory flow through decreasing endothelial edema (53), and combating inflammation through suppression of pro-inflammatory mediators and increased anti-inflammatory mediators (54, 55). Despite these favorable attributes, a multicenter randomized control study on hemorrhagic shock comparing normal saline, hypertonic saline, and hypertonic saline dextran did not show any difference in survival (56). Subsequent to this study, interests in hypertonic saline in prehospital resuscitation of hemorrhagic shock has decreased as evidence for more favorable outcomes using plasma for volume replacement has become evident.

Synthetic colloids such as hydroxyethyl starch (HES) have historically been used as a resuscitative fluid in hemorrhagic shock patients in veterinary medicine due to their rapid volume expansion, availability and cost (57, 58). Recent evaluation of their effects on coagulation in canine hemorrhage models (33) and attenuation of their use in human clinical medicine has impacted veterinary utilization (*cross reference Frontiers article: Colloids…“Article 7 Colloids, Yes or No? Pros and Cons of Colloids?” (Adamik/Yosova)*).

### Permissive Hypotension

Similar to the philosophical approach to damage control surgery (DCS) where there is an attempt to stop or minimize hemorrhage as soon as possible in the surgical suite, anecdotal clinical observations of increasing blood pressure with fluid resuscitation may create vascular pressure exacerbated bleeding. To this end, studies were developed to investigate the effects of minimizing increases in blood pressure to allow for vital organ perfusion but limited excessive pressures beyond that. Many of the initial studies were conducted in animal models and a summary of the results was published in a meta-analysis of these studies (59). All resulted in decreased mortality compared to normotensive fluid resuscitation. Taking these results to the human field, the results have been less conclusive. One study compared normal pre-hospital fluid resuscitation to delaying fluid resuscitation in penetrating torso patients until arrival in the trauma operating room. They found improved survival in the latter group (70 vs. 62%) (60). Another study compared high blood pressure (systolic: 100 mmHg) to low blood pressure (systolic: 70 mmHg) endpoints and there was no difference in mortality (61). A subsequent study with similar systolic pressure endpoints reported improved survival in blunt trauma patients but not in penetrating trauma patients (62).

There is not strong evidence to support an ideal pressure goal with hypotensive resuscitation and how long it can be maintained. In addition, most of these studies have used crystalloid solutions as part of their resuscitation and there have been no definitive studies using hypotensive resuscitation with blood component therapy. None of the studies cited above were performed in patients with head trauma and hypotensive resuscitation in patients with head trauma and increased intracranial pressure is not recommended (63, 64).

### Component Therapy

Early studies on animal blood transfusion demonstrated the effectiveness of whole blood transfusion on resuscitation in hemorrhagic shock but it was fraught with infections, clotting and other complications. With refinement in collection techniques and anti-coagulants, these hurdles were overcome and whole blood transfusion became a useful therapy in hemorrhagic shock. The scarcity of blood products eventually led to the more efficient use of component therapy.

Early standard resuscitation component therapy included sequential resuscitation with crystalloids, artificial colloids, and packed red blood cells (65). With the advent of Damage Control Resuscitation (DCR), this type of component therapy began to evolve to an increasing plasma to packed red blood cell (pRBC) ratio. A study performed during the Iraq War investigated the use of three different patient cohorts based on ratios of plasma:pRBC including a low ratio (1:8), a medium ratio (1:2.5) and a high ratio (1:1.4) (66). The all-cause mortality and death from hemorrhage rates both decreased in the low ratio cohort when compared to the other two cohorts. Similar findings were demonstrated in people with ruptured aortic aneurysm using historical controls (67). Another observational study in civilian trauma patients showed similar findings with early resuscitation with higher ratios of plasma and platelets to pRBCs (68). More evidence supporting higher ratios of plasma and platelets was demonstrated in a prospective, randomized clinical trial in bleeding trauma patients comparing 1:1:1 to 1:1:2 plasma to platelet to pRBCs (69). Although no difference in 24-h or 30-day survival was noted, the lower ratio group was more likely to achieve hemostasis and had decreased death due to hemorrhage. These studies and others have led to the recommendations using higher plasma to pRBC ratios during massive transfusion (> 10 units of packed red blood cells within a 24 h period or > 5 units within 4 h). In fact, in the American College of Surgeons Trauma Quality Improvement Program Massive Transfusion in Trauma Guidelines, developed by a panel of expert trauma surgeons, recommend to begin resuscitation with blood component therapy (rather than using crystalloids or colloids) with a plasma to pRBC ratio ranging from 1:1 to 1:2 ratio (70).

The underlying benefits using component therapy compared to crystalloid resuscitation are not completely elucidated but several reported advantages include but are not limited to mitigation of hyperfibrinolysis and platelet function and repair of endothelial integrity (71, 72). In veterinary medicine, utilization of (fresh) frozen plasma and stored pRBCs, in a 1:1 or 1:2 ratio is feasible.

### Platelets

Given the improvement in outcomes using plasma and blood component therapy studies have been performed to investigate the use of platelets in blood component therapy. Similar to plasma, high ratios of platelet concentrates to pRBC have demonstrated decreased risk of death in massively transfused patients (68, 73, 74). The American College of Surgeons Committee on Trauma recommends transfusing one unit of platelets to every 6 units of pRBCs (70).

### Fresh Whole Blood

Given the success of component therapy, why not just use fresh whole blood? The use of fresh whole blood in war casualties has an extensive history particularly from the conflicts in Afghanistan and Iraq (4). Studies during these conflicts found improved survival in patients that received fresh whole blood compared to plasma and pRBC component therapy. A particular advantage of fresh whole blood is that it does not require drawing multiple component units and provides balanced components in one administration. There are many hurdles to the use of fresh whole blood including access, the short shelf life of “fresh whole blood” and rapid screening for infectious disease (unless donor present has already been screened) (75–77).

### The Veterinary Caveat

Component therapy has become the preferred method in resuscitation of severe hemorrhage in humans but there are hurdles to the use of it in veterinary medicine. Many of the studies cited in this section above indicated that this therapy is most effective within the first few hours of hemorrhage and its effect on outcome diminishes after this period. Anecdotally, it is recognized that the speed with which hemorrhaging veterinary patients are taken to surgery for definitive hemorrhage control is much more delayed compared to human medicine (with some exceptions). This delay may negate the positive results with component therapy. Similarly, component therapy may not be readily available to the many veterinary hospitals. Additionally, plasma concentrates are also not consistently available. While there remains much to learn in clinical veterinary patients, experimental studies in animals show that replacement of lost blood, sometimes as components, administered early has improved outcomes (18, 20, 23).

## Current Recommendations

Consensus on the best approach to volume resuscitation in the acutely hemorrhaging patient does not exist in veterinary medicine due to multiple factors including, but not limited to, lack of prospective clinical trials, limited similarly designed projects amenable to meta-analyses, and challenges extrapolating human and pre-clinical model findings to clinical canine and feline patients with similar, but not identical, physiologic responses to hemorrhage and resuscitation. However, this challenge is not unique to veterinary medicine, and continued research efforts to individualize resuscitation in the hemorrhaging patient based on patient phenotype, response to interventions, degree of disease and environment remain a significant pursuit (3, 12, 78). In the meantime, clinicians are left to make decisions for patients daily based on interpretation of the research that exists. The following recommendations regarding the approach to the acutely hemorrhaging canine or feline trauma patient relies heavily on the authors' interpretation of preclinical, mechanistic and limited veterinary clinical studies reviewed in the references. The algorithm presented here for veterinary patients was inspired by and adapted from a similar algorithm developed by the ATOMAC group regarding non-operative management of blunt liver and spleen injury in children (79). The authors put this forth as a current recommended guideline, and anticipate generation of a similar guideline in the near future as veterinary groups begin developing clinical practice guidelines based using GRADE methodology. Evidence to support best practices for volume resuscitation in the hemorrhaging cat is sparse, and efforts to acknowledge differences are made.

Initial triage of the acutely ill patient has many approaches with different assessment algorithms taught and applied. The British military has added Catastrophic bleeding (< C>) to the American College of Surgeons Airway (A), Breathing (B), Circulation (C), Disability (D), Environment/Exposure (E) approach to the primary survey of the acutely injured patient methodology that it advocates utilizing (80). Regardless of approach, patients with circulatory shock associated with blood loss require immediate intravascular access and interventions to ensure adequate delivery of oxygen to tissues. While determination of an underlying cause is important, respiratory, cardiovascular, and neurologic stabilization should be prioritized and initiated immediately.

**Figure 3:**
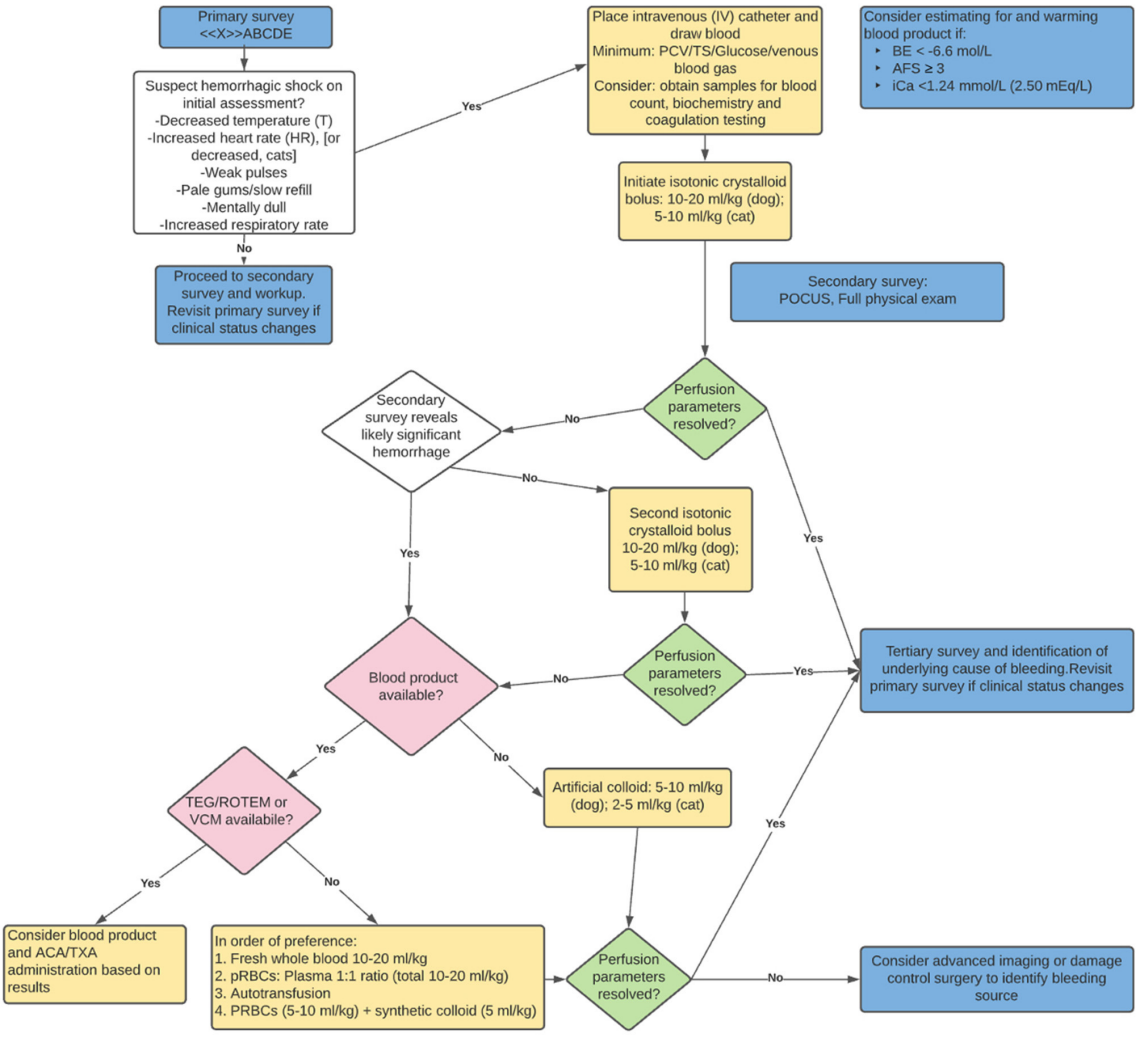
Recommended resuscitation approach for the acutely hemorrhaging patient. BE, base excess; AFS, abdominal fluid score; iCa, ionized calcium; POCUS, point of care ultrasound; TEG, thromboelastography; ROTEM, rotational thromboelastometry; VCM, viscoelastic coagulation monitoring; ACA, aminocaproic acid; TXA, tranexamic acid; pRBC, packed red blood cells.

During the intravascular resuscitation phase of the acutely hemorrhaging patient, reassessment parameters include physical exam findings (temperature, respiratory rate and effort, pulse rate and quality, mucous membrane color, capillary refill time, and mentation), biochemical measurements [packed cell volume or hemoglobin, total protein/solids, base excess (deficit), lactate and pH], and macro-perfusion measurements (blood pressure). Once circulatory stabilization is underway, continued determination of underlying cause (Table 1) and consequences to determine further intervention is required. If perfusion parameters from physical exam, biochemical measurements, or macro-perfusion measurements worsen during resuscitation, reassessment of the primary survey is initiated, and interventions selected based on patient findings and patient status at that point (Figure 3).

While there continues to be a shift in first choice resuscitation in the recently hemorrhaged patient in shock, current literature (and logistics) supports a 10–20 ml/kg dog (5–10 ml/kg cat) intravascular dose of a balanced electrolyte solution (LRS, Plasma-lyte 148, Normosol-R). In the subset of hemorrhaging patient's whose clinical status has not stabilized, the clinician's choices from there vary based on patient status, response to interventions and blood product availability. Consideration for blood product administration in the poorly responding hemorrhaging patient early in resuscitation is recommended (Figure 3).

A series of veterinary papers evaluating various triage values and subsequent blood transfusion in canine trauma patients found an association between abdominal fluid score (AFS), base excess (BE) and ionized calcium (iCa), respectively, as independent predictors of patients receiving a blood transfusion. These findings suggest consideration for readying blood product for administration when initial triage diagnostics include an AFS ≥ 3, BE < − 6.6 mmol/L, and ionized iCa < 1.24 mmol/L or < 2.50 mEq/L (30, 81, 82). Given the logistics required to warm (pRBCs) and/or thaw (FFP), when these parameters are identified, the recommendation is to considering beginning that process.

Veterinary and human literature demonstrate a spectrum of coagulation and fibrinolytic abnormalities in acutely hemorrhaging patients (3, 9–11, 34). In human medicine, antifibrinolytic agents have been studied and utilized in various clinical scenarios that result in hemorrhage, including trauma, postpartum and orthopedic surgery patients (83). The CRASH-2 trial demonstrated improved survival in acutely hemorrhaging trauma patients when tranexamic acid (TXA) is administered within 3 h of injury (84). In veterinary medicine, there is evidence that dogs are more hyperfibrinolytic when compared to humans (85). While numerous studies support the effectiveness of TXA in blunting hyperfibrinolysis in dogs (86, 87), unless viscoelastic testing evidence of hyperfinbrinolysis resulting in ongoing bleeding is evident, its “standard use” in all hemorrhaging patients has not yet been established (88). That said, given limited availability to universally available viscoelastic testing in veterinary medicine, in an acutely hemorrhaging canine patient not responding to hemorrhage control and volume resuscitation, it is reasonable to consider the use of antifibrinolytics (TXA or Aminocaproic acid). While the literature is sparse, cats may be at more risk for developing comorbidities with administration of TXA (89). Additionally, there is risk of hypercoagulability and thromboembolic events in cats after trauma (90). The routine utilization of antifibrinolytics in hemorrhaging cats is not recommended based on current literature.

In summary, current evidence supports judicious use of a balanced electrolyte solution, and early movement to “replacing what is lost” in the acutely hemorrhaging patient by utilization of blood product, when available (Figure 3). Future developments in lyophilized or other “long shelf life” component therapy may help bridge the gaps in cost, access and availability of blood product in veterinary medicine, and if research supports their effectiveness, may expand veterinarian's ability to address the hemorrhaging patient more universally.

## Author Contributions

All authors listed have made a substantial, direct and intellectual contribution to the work, and approved it for publication.

## Conflict of Interest

The authors declare that the research was conducted in the absence of any commercial or financial relationships that could be construed as a potential conflict of interest.

## Publisher's Note

All claims expressed in this article are solely those of the authors and do not necessarily represent those of their affiliated organizations, or those of the publisher, the editors and the reviewers. Any product that may be evaluated in this article, or claim that may be made by its manufacturer, is not guaranteed or endorsed by the publisher.
